# Intravascular ultrasound guidance in drug-eluting stents implantation: a meta-analysis and trial sequential analysis of randomized controlled trials

**DOI:** 10.18632/oncotarget.19613

**Published:** 2017-07-27

**Authors:** Cheng Qian, Hong Feng, Jianlei Cao, Guangyu Zhang, Yanggan Wang

**Affiliations:** ^1^ Department of Cardiology, Zhongnan Hospital of Wuhan University, Wuhan University, Wuhan 430071, China

**Keywords:** intravascular ultrasound, drug-eluting stents, meta-analysis, trial sequential analysis, randomized controlled trials

## Abstract

**Objective:**

Previous evidence suggested that intravascular ultrasound (IVUS) guidance could improve outcomes after drug-eluting stents (DES) placement, largely driven by data from observational studies. We, therefore, performed a meta-analysis and trial sequential analysis of randomized controlled trials to overcome this limitation.

**Results:**

The retrieval process yielded 7 RCTs with 3,192 patients. Compared to the angiography guidance, IVUS-guided DES implantation was associated with a significant reduction in the major adverse cardiac events (MACE) (OR 0.60, 95% CI 0.46-0.78; *P* < 0.001), target vessel revascularization (OR 0.60, 95% CI 0.40-0.91; *P* = 0.02) and target lesion revascularization (OR 0.60, 95% CI 0.42-0.85; *P* = 0.004). IVUS and conventional angiography guidance showed similar incidence of stent thrombosis (ST) (OR 0.56, 95% CI 0.25-1.23; *P* = 0.15), cardiac death (OR 0.47, 95% CI 0.19-1.15; *P* = 0.10) and myocardial infarction (OR 0.85, 95% CI 0.45-1.61; *P* = 0.62). Trial sequential analysis revealed a definite reduction in MACE with IVUS guidance without solid evidence for ST.

**Materials and Methods:**

A systematical literature search was performed in the databases of PubMed, the Cochrane Library and ClinicalTrials.gov, complemented with reference screening from relevant articles. Primary endpoints were MACE and ST.

**Conclusions:**

IVUS-guided DES implantation is associated with a lower risk of MACE and revascularization without conclusive benefits for ST.

## INTRODUCTION

Drug-eluting stents (DES) are widely used as a great advance in the interventional cardiology. However, some drawbacks remain in its routine clinical use, including the risk of in-stent restenosis and stent thrombosis that are striking in complex lesion morphology [1–3]. Intravascular ultrasound (IVUS), a catheter-based invasive imaging technique with high solution, appears to be useful for precise evaluation of coronary anatomy, lesion characteristics, optimal stent implantation, and potential complications after stent deployment [4–6].

Several previous meta-analyses showed that IVUS-guided DES implantation was associated with favorable outcomes compared to the angiography guidance [7–11]. However, these findings were mostly driven by data from observational studies. Recently, 4 additional randomized controlled trials (RCTs) have been published. We, therefore, conducted a meta-analysis of RCTs to further investigate the clinical outcomes of IVUS- versus angiography-guided strategies in DES implantation.

## RESULTS

**Figure 1:**
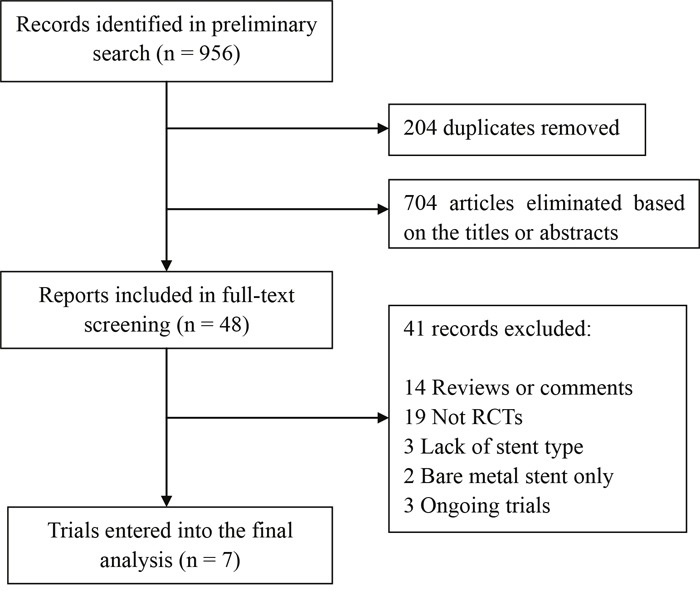
Flow diagram of data search

Of the initial 956 reports, 204 were duplicates, and 704 were excluded based on the title or abstract. The remaining 48 studies underwent full-text screening, of which 41 records failed to meet the inclusion criteria and therefore were excluded. Finally, 7 RCTs [12–18] conducted from 2010 to 2015, comprising a total of 3,192 patients, entered into the final analysis (Figure 1).

The main characteristics of the included trials are summarized in Table 1. In general, 1,593 patients were assigned to the IVUS-guided group and 1,599 patients to the angiography-guided group, with a weighted clinical follow-up of 14.8 months. Cochrane risks of bias assessment revealed a high methodological quality (Supplementary Figure 1). Table 2 exhibited the principal characteristics of the participants. The patients' mean age ranged from 59 to 77 years old, and men accounted for 71% of the total patients. Nearly 41% of the patients were presented with acute coronary syndrome. Baseline angiographic and procedural characteristics were shown in Supplementary Table 1.

**Table 1:** Characteristics of the included trials

Study	Publication Year	Multicentre	Sample Size	New-generation DES (%)	Treated Lesion	Primary Endpoint	Angiographic F/U (months)	Clinical F/U (months)
AVIO [12]	2013	Yes	142/142	NA	Complex	Minimal lumen diameter	Various	24
CTO-IVUS [13]	2015	Yes	201/201	100/100	CTO	Cardiac death	NA	12
HOME DES IVUS [14]	2010	No	105/105	0/0	Complex	Death, MI, and TLR	NA	18
IVUS-XPL [15]	2015	Yes	700/700	100/100	Long	Cardiac death, target lesion-related MI, and ID-TLR	NA	12
RESET [16]	2013	Yes	269/274	100/100	Long	Cardiac death, MI, ST, and TVR	Not routine	12
Tan et al [17]	2015	No	61/62	0/0	ULMCA	Death, non-fatal MI, and TLR	9-12	24
Tian et al [18]	2015	Yes	115/115	28/20	CTO	Late lumen loss	12	24

CTO, chronic total occlusion; DES, drug-eluting stent; F/U, follow-up; ID-TLR, ischemia-driven target lesion revascularization; MI, myocardial infarction; NA, not applicable; ST, stent thrombosis; TLR, target lesion revascularization; TVR, target vessel revascularization; ULMCA, unprotected left main coronary artery.

**Table 2:** Characteristics of the patients enrolled in the included trials

Study	Age(years)	Male(%)	Hypertension(%)	DM(%)	Smoker(%)	LVEF(%)	Prior MI(%)	Prior PCI(%)	ACS(%)	MutivesselDisease (%)
AVIO [12]	64/64	82/77	70/67	24/27	35/31	55/56	NA	NA	30/26	NA
CTO-IVUS [13]	61/61	81/81	63/64	35/34	35/34	57/57	8/8	15/16	0/0	72/63
HOME DES IVUS [14]	59/60	73/71	67/71	42/45	40/35	NA	37/32	17/14	62/60	60/54
IVUS-XPL [15]	64/64	69/69	65/63	36/37	22/26	63/62	5/4	11/10	49/49	67/70
RESET [16]	63/64	66/55	61/66	32/30	22/17	55/54	1/3	NA	47/49	41/38
Tan et al [17]	77/76	62/69	41/47	34/30	44/47	55/53	16/21	NA	70/66	84/89
Tian et al [18]	67/66	89/80	75/70	30/27	39/39	55/56	21/30	20/21	29/24	85/83

ACS, acute coronary syndrome; DM, diabetes mellitus; LVEF, left ventricular ejection fraction; MI, myocardial infarction; NA, not applicable; PCI, percutaneous coronary intervention.

### MACE and ST

**Figure 2:**
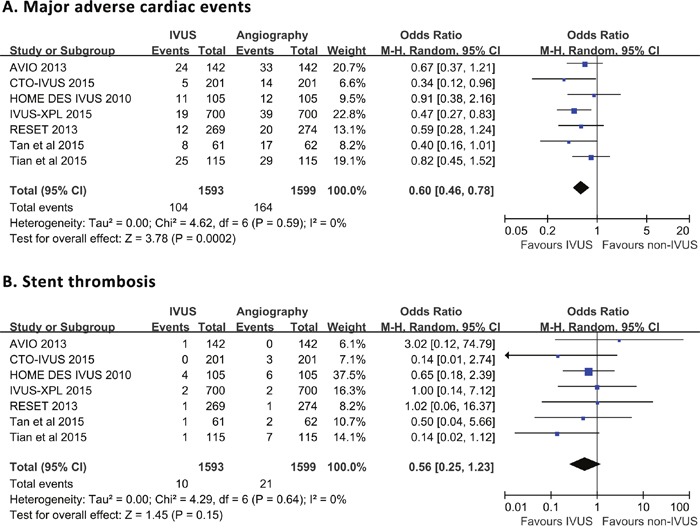
Comparison of IVUS-guided and angiography-guided DES implantation for the risk of major adverse cardiac events **(A)** and stent thrombosis **(B)**.

Meta-analytic pooling for primary outcomes was shown in Figure 2. Briefly, MACE was recorded in 104 (6.5%) and 164 (10.3%) patients in the IVUS- and angiography-guided groups, respectively, without significant heterogeneity across trials (*P* = 0.59, *I*^2^ = 0%). Compared with angiography guidance, IVUS-guided DES implantation was associated with a significant reduction in the risk of MACE (OR 0.60, 95% CI 0.46-0.78, *P* < 0.001; NNT 27, 95% CI 18-55). Definite/probable ST occurred in 10 (0.6%) and 21 (1.3%) individuals with DES implantation guided by IVUS and angiography, respectively. There was no considerable heterogeneity across the trials (*P* = 0.64, *I*^2^= 0%). Pooled data indicated that thrombosis risk was similar between IVUS- and angiography-guided groups (OR 0.56, 95% CI 0.25-1.23, *P* = 0.15).

### Revascularization, cardiac death, and MI

**Figure 3:**
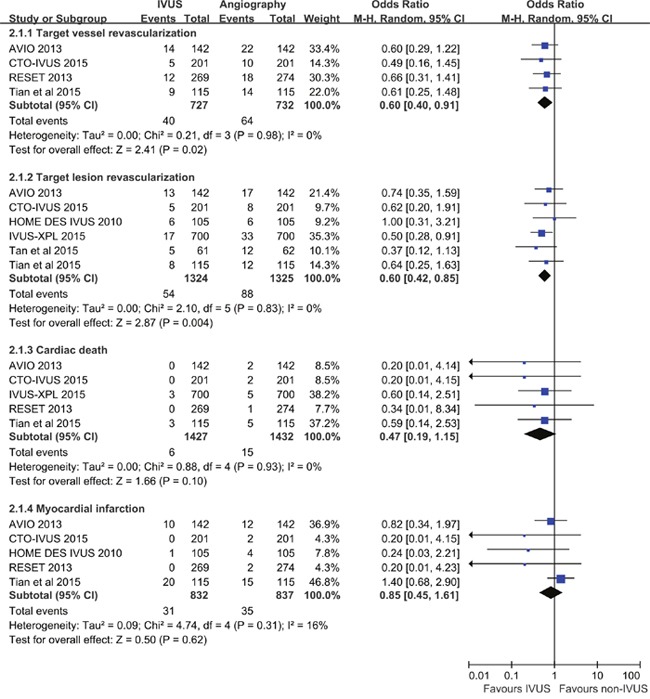
Comparison of IVUS-guided and angiography-guided DES implantation for the risk of target vessel revascularization, target lesion revascularization, cardiac death, and myocardial infarction

Figure 3 showed summarized ORs for the secondary outcomes, and no significant heterogeneity has been detected (TVR: *P* = 0.98, *I*^2^ = 0%; TLR: *P* = 0.83, *I*^2^ = 0%; cardiac death: *P* = 0.93, *I*^2^ = 0%; MI: *P* = 0.31, *I*^2^ = 16%). Compared to angiography guidance, IVUS guided-DES implantation resulted in a significantly lower risk of TVR (OR 0.60, 95% CI 0.40-0.91, *P* = 0.02; NNT 31, 95% CI 17-172) and TLR (OR 0.60, 95% CI 0.42-0.85, *P* = 0.004; NNT 39, 95% CI 23-118). The risk of cardiac death and MI in the IVUS-guided group was comparable to those in the angiography-guided group (cardiac death: OR 0.47, 95% CI 0.19-1.15, *P* = 0.10; MI: OR 0.85, 95% CI 0.45-1.61, *P* = 0.62).

### Procedural parameters

The meta-analyses of procedural parameters were shown in Supplementary Figure 2. Between IVUS-guided and angiography-guided groups, there was no difference in the stent lengths (WMD 1.21 mm, *P* = 0.07) and diameters (WMD 0.07 mm, *P* = 0.09). However, IVUS-guided PCI was associated with higher maximal inflation pressure (WMD 0.66 atm, *P* = 0.004), larger MLD (WMD 0.08 mm, *P* < 0.001), and slighter DS (WMD -1.25%, *P* < 0.001) compared with the angiography-guided PCI.

### Sensitivity analysis

For the primary outcomes, no influence from single study or application of fixed-effect model was detected. Similarly, the pooled estimate of MACE remained significant after the introduction of Knapp-Hartung modification (OR 0.60, 95% CI 0.45-0.80, *P* = 0.005). In addition, the pooled ORs for both MACE and ST were neither associated with the weight of disease, e.g. acute coronary syndrome and diabetes, nor the chronic total occlusion lesions, the use of new-generation DES implanted, the mean baseline age and the stent length (Supplementary Table 3).

### Publication bias

No funnel plot for primary outcomes was skewed through visual judgment, suggesting the absence of small-study effect. Additionally, neither Egger's test nor Begg's test showed the potential for publication bias (Supplementary Figure 3).

### Trial sequential analysis

**Figure 4:**
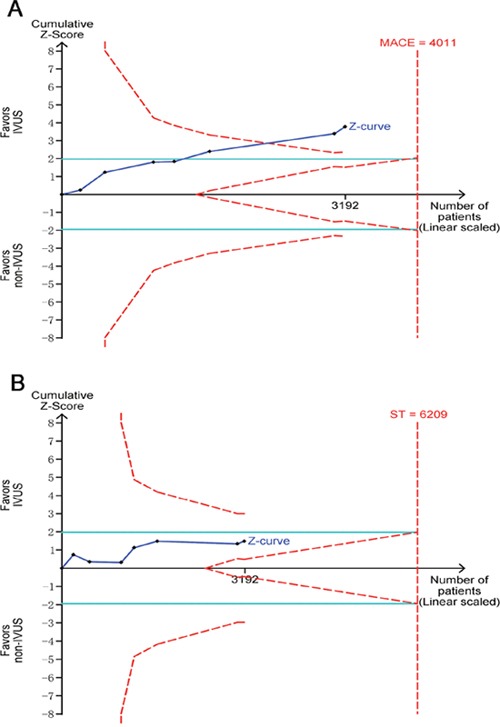
Trial sequential analyses for major adverse cardiac events **(A)** and stent thrombosis **(B)**.

Trial sequential analysis for the evaluation of MACE revealed that 79.6% of the required sample size (4,011 patients) was accrued in the current analysis. The cumulative Z curve crossed the boundaries for superiority, further confirming the pronounced reduction in the incidence of MACE associated with IVUS guidance (Figure 4A). For the assessment of ST, however, only 51.4% of the estimated sample size (6,209 patients) was accrued. The Z curve did not cross any monitoring boundaries, indicating an inadequate power for making a clear conclusion upon this endpoint (Figure 4B).

## DISCUSSION

In the present meta-analysis of 7 RCTs comprising a total of 3,192 patients, we found that compared with angiography guidance, IVUS-guided DES implantation was associated with a reduced risk of MACE, TVR, and TLR. The incidence of ST, cardiac death, and MI were comparable between IVUS and angiography guidance. DES implantation under IVUS guidance also showed improvement in procedural angiographic parameters. In addition, trial sequential analysis reflected a definite reduction in MACE with IVUS guidance, without firm evidence for ST.

Although some guidelines give a class IIa recommendation for IVUS use in selected patients to optimize stent implantation, the evidence on which it was based were data from registries or RCTs in pre-DES era [19]. Currently, DES is widely used in clinical practice, thus powerful evidence is need to confirm the outcomes of IVUS-guided DES implantation. The findings of our study are consistent with previous meta-analyses, which showed that IVUS guidance could reduce the risks of MACE and repeat revascularization following DES implantation [9–11]. Noteworthy, these studies included both randomized trials and observational studies, leading to somewhat bias because of impossibility to fully eliminate interference from residual confounding factors. The present work enrolled addtional 4 recent RCTs [13, 15, 17, 18], which represent a more reliable and comprehensive assessment of the clinical impact of IVUS guidance on DES implantation. The benefits of IVUS-guided stening are also indicated in a recent meta-analysis of RCTs [20]. However, that study did not perform important additional analyses, such as trial sequetial analyses, to confirm their findings.

The beneficial effect on MACE in our meta-analysis may be associated with the lower rate of repeat revascularization and the numerically reduced cardiac death in the IVUS-guided group. Increasing evidence suggests that a large post-interventional MLD may serve as an important contributor to preventing restenosis following DES implantation [21], and the predictive value of DS to in-stent restenosis has also been reported [22]. In the present study, both post-PCI MLD and DS were significantly improved in the IVUS-guided compared to the angiography-guided groups. Theoretically, this may contribute to the reduced risk of repeat revascularization and, consequently, the composite endpoint MACE. However, some included studies that showed a greater MLD after IVUS-guided DES placement did not show an expected decrease in MACE or revascularization events [12, 18]. The seeming paradox indicates that other factors may also contribute to the observed benefits associated with IVUS use. For example, IVUS imaging for evaluation of stenosis severity has shown excellent correlation with fractional flow reserve, which represents the criterion standard to assess the prognostic value of coronary stenosis [23].

IVUS guidance is useful for identifying mechanical factors for ST events, including stent underexpansion, stent edge dissection, and incomplete stent apposition [24, 25]. At variance with other relevant meta-analyses, the current data showed that IVUS guided-PCI was not correlated with a reduced risk of ST and MI. A possible explanation for the divergence is that most of patients in our study received new-generation DES (approximately 75%), while these devices were much less implanted in the patients enrolled in the earlier meta-analyses. Although it may not affect the pooled OR for ST, the use of new-generation DES significantly reduced the ST events [26] as shown by the insufficient statistical power for ST in our trial sequential analysis. Along this line, the largest RCT [15] investigating the utility of IVUS guidance in everolimus-eluting stents implantation also failed to establish a marked difference in ST risk (hazard ratio, 1.00; *P* > 0.99). Moreover, there were no standard criteria for optimal stenting under IVUS guidance among the included trials (Supplemental Table 1). Whether it is also related to the ST events remains in question.

There are some limitations in our study. First, most of the included RCTs enrolled highly selected patients with complex lesions. Thus, extrapolating our findings to patients with simple lesions deserves cautions. Second, blind design was not adopted in all included trials because of the obvious difference in IVUS- and angiography-guided procedures. As such, performance bias cannot be eliminated completely. Third, the follow-up duration of our meta-analysis is relatively short; long-term data are needed to further confirm the benefits associated with IVUS-guided PCI. Fourth, owing to the lack of separate data, we cannot accurately evaluate the influence of baseline characteristics (clinical presentation, lesion feature, etc.) on the benefit of IVUS guidance. Fifth, the criteria for IVUS guidance are distinct across the included studies, which may affect the final results of our meta-analysis. Finally, meta-regression analysis may be not powered because only 7 clinical trials were included in the present meta-analysis.

In conclusion, our study indicates that compared to angiography guidance, IVUS-guided DES implantation is associated with lower risks of MACE and repeat revascularization, without difference in cardiac death and MI. In addition to these positive findings, it should be noted that all the trials were performed by operators expert in intracoronary imaging. Therefore, we still need evidence supporting routine use of IVUS-guided PCI in further dedicated randomized trials.

## MATERIALS AND METHODS

### Literature sources

This study was performed in accordance with the Preferred Reporting Items for Systematic Reviews and Meta-Analyses statement [27]. We performed a systematic literature search in databases of PubMed, the Cochrane Library, and CinicalTrials.gov from January 2000 to December 2015 to identify eligible records. The reference lists of all relevant reviews and meta-analyses were also scanned for more trials. The search terms were as follows: “intravascular ultrasound”, “IVUS”, “IVUS-guided”, “IVUS guidance”, “angiography”, “drug-eluting stent”, and “percutaneous coronary intervention (PCI)” (seeing detailed search strategy in Supplementary Table 2).

### Eligibility criteria

To be included, the potentially eligible studies should meet the following requirements: 1) RCTs comparing IVUS guidance with coronary angiography guidance for DES implantation, 2) with a clinical follow-up duration ≥ 6 months, and 3) availability of outcomes that were investigated in this meta-analysis. Studies without a report on stent types (bare metal stent or DES) were discarded.

### Data extraction and quality assessment

The details on study and patient characteristics were extracted. Quality judgment for included trials was performed by using the Cochrane collaboration's tool for assessing the risk of bias [28] with the following 6 main domains: random sequence generation, allocation concealment, binding of participants and personnel, binding of outcomes assessment, incomplete outcome data, and selective reports. Data extraction and quality assessment were conducted by 2 independent reviewers, with consensus achieved by discussion with a third reviewer.

### Endpoints

The primary endpoints of this meta-analysis were the major adverse cardiac event (MACE) and stent thrombosis (ST). As the definitions of MACE were not completely uniform across the 7 trials, we used the study-specific definitions. ST was defined according to the Academic Research Consortium criteria [29]. Secondary outcomes included target vessel revascularization (TVR), target lesion revascularization (TLR), cardiac death, and myocardial infarction (MI). Event rate was evaluated at the longest available follow-up based on the intention-to-treat analysis. The largest RCT by Hong et al. [15] did not report TLR, thus we extracted the ischemic-driven TLR instead. Procedural parameters were also assessed, including stent length and diameter, maximal inflation pressure, minimal lumen diameter (MLD), and diameter stenosis (DS).

### Statistical analysis

Odds ratio (OR) with corresponding 95% confidence interval (CI) was used as pooled statistics for categorical outcomes, while weighted mean differences (WMD) was used to represent the estimates for continuous variables. The pooled estimate was calculated under random effects model with DerSimonian-Laird method. A number-needed-to-treat (NNT) statistic was also estimated in the case of significant results. Heterogeneity across studies was explored by the Cocharane Q test with a significant level of *P* < 0.1. In addition, we used the *I*^2^ statistic to describe the quantification of heterogeneity, with an *I*^2^ value > 25% considered as substantial heterogeneity. The robustness of the summarized estimates was evaluated for primary outcomes by sensitivity analysis, including one-at-a-time trial exclusion and applications of fixed-effect model or random-effect model with Knapp-Hartung modification. Again, we did meta-regression analyses to identify covariates that might influence the risk estimates of primary endpoints, including the percentage of patients with acute coronary syndrome and diabetes, the portion of chronic total occlusion treated, the frequency of new-generation DES used, baseline mean age, and stent length. Publication bias was appraised by visual inspection of funnel pots and the Egger's and Begg's tests. We set the *P* value threshold for significance at 0.05. Data analysis was performed using Review Manager 5.2 (RevMan, The Cochrane Collaboration, Copenhagen, Denmark) and STATA 12.0 software (STATA Corp, College Station, TX, USA).

### Trial sequential analysis

Trial sequential analysis was conducted for assessing the accumulating evidence and sample size [30], by using the TSA 0.9 Beta (available at http://www.ctu.dk/tsa). Our assumptions consisted of 2-sided testing, type I error of 5% and power of 80%. We tested the hypothesis that IVUS guidance could yield a 25% and 50% relative reduction in the risk of MACE and ST, respectively, with an anticipated event rate of 10% for MACE and of 1.5% for ST in the angiography-guided group. The main results were displayed in a graph of the cumulative Z curve, and the O'Brien-Fleming α-spending function was used to determine the boundaries in this graph for concluding superiority, inferiority, or non-inferiority.

## SUPPLEMENTARY MATERIALS FIGURES AND TABLES


