# Supplementary material for: Intravascular ultrasound guidance in drug-eluting stents implantation: a meta-analysis and trial sequential analysis of randomized controlled trials
Source: Oncotarget. 2017 Jul 27;8(35):59387–96. doi: 10.18632/oncotarget.19613 (PMC5601740; doi:10.18632/oncotarget.19613)
Supplement: Supplementary file 1 [file oncotarget-08-59387-s001.pdf]

Intravascular ultrasound guidance in drug-eluting stents implantation: a meta-analysis and trial sequential analysis of randomized controlled trials

SUPPLEMENTARY MATERIALS

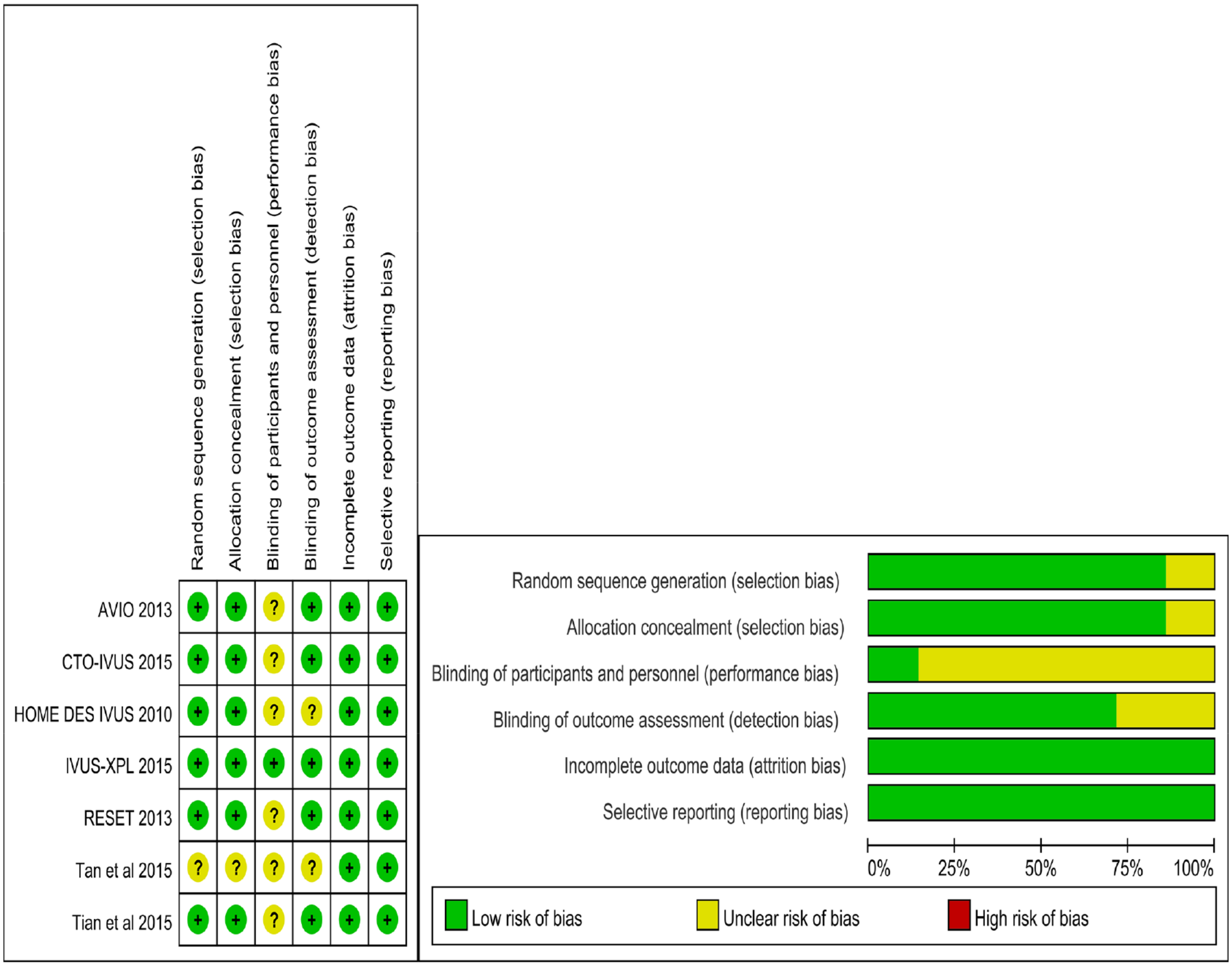

Supplementary Figure 1: Cochrane risks for bias assessment.

A. Stent length

Study or Subgroup	IVUS Mean	IVUS SD	IVUS Total	Angiography Mean	Angiography SD	Angiography Total	Weight	Mean Difference IV, Random, 95% CI
AVIO 2013	23.9	6.74	142	23.2	6.51	142	29.5%	0.70 [-0.84, 2.24]
CTO-IVUS 2015	43.6	18.7	201	41.5	17.6	201	10.7%	2.10 [-1.45, 5.65]
IVUS-XPL 2015	39.3	13.1	700	39.2	12.3	700	32.9%	0.10 [-1.23, 1.43]
Tan et al 2015	21.48	6.37	61	18.24	4.88	62	22.9%	3.24 [1.23, 5.25]
Tian et al 2015	55	23	115	55	25	115	4.1%	0.00 [-6.21, 6.21]
**Total (95% CI)**			**1219**			**1220**	**100.0%**	**1.21 [-0.10, 2.51]**

Heterogeneity: Tau² = 0.89; Chi² = 7.15, df = 4 (P = 0.13); I² = 44%
Test for overall effect: Z = 1.81 (P = 0.07)

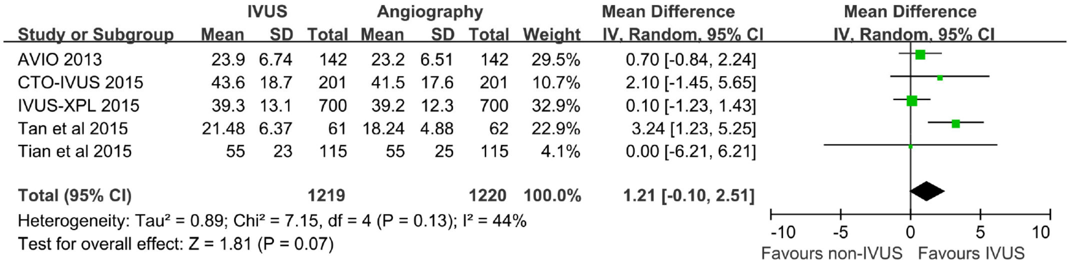

B. Stent diameter

Study or Subgroup	IVUS Mean	IVUS SD	IVUS Total	Angiography Mean	Angiography SD	Angiography Total	Weight	Mean Difference IV, Random, 95% CI
AVIO 2013	2.95	0.38	142	2.86	0.36	142	24.4%	0.09 [0.00, 0.18]
CTO-IVUS 2015	2.91	0.52	201	2.85	0.41	201	23.7%	0.06 [-0.03, 0.15]
Tan et al 2015	3.43	0.09	61	3.44	0.12	62	30.5%	-0.01 [-0.05, 0.03]
Tian et al 2015	3.05	0.46	115	2.86	0.37	115	21.4%	0.19 [0.08, 0.30]
**Total (95% CI)**			**519**			**520**	**100.0%**	**0.07 [-0.01, 0.16]**

Heterogeneity: Tau² = 0.01; Chi² = 15.07, df = 3 (P = 0.002); I² = 80%
Test for overall effect: Z = 1.69 (P = 0.09)

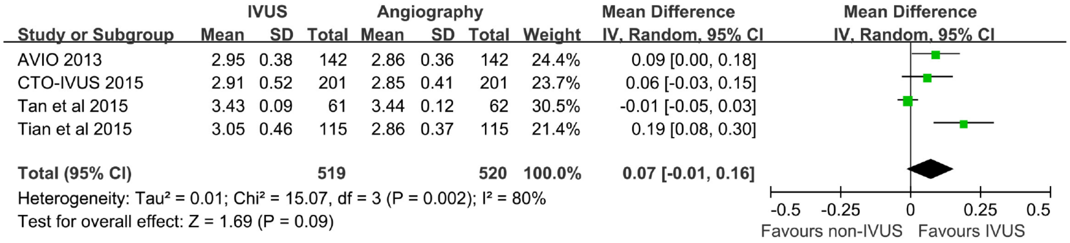

C. Maximal inflation pressure

Study or Subgroup	IVUS Mean	IVUS SD	IVUS Total	Angiography Mean	Angiography SD	Angiography Total	Weight	Mean Difference IV, Random, 95% CI
AVIO 2013	20.3	4.82	142	19.6	4	142	11.8%	0.70 [-0.33, 1.73]
CTO-IVUS 2015	14.6	3.7	201	13.8	3.8	201	17.2%	0.80 [0.07, 1.53]
HOME DES IVUS 2010	16.4	1.7	105	15.2	1.5	105	24.6%	1.20 [0.77, 1.63]
IVUS-XPL 2015	16.5	4.1	700	15.9	4.1	700	24.7%	0.60 [0.17, 1.03]
RESET 2013	13.5	3.3	269	13.5	3.1	274	21.8%	0.00 [-0.54, 0.54]
**Total (95% CI)**			**1417**			**1422**	**100.0%**	**0.66 [0.22, 1.11]**

Heterogeneity: Tau² = 0.16; Chi² = 11.87, df = 4 (P = 0.02); I² = 66%
Test for overall effect: Z = 2.91 (P = 0.004)

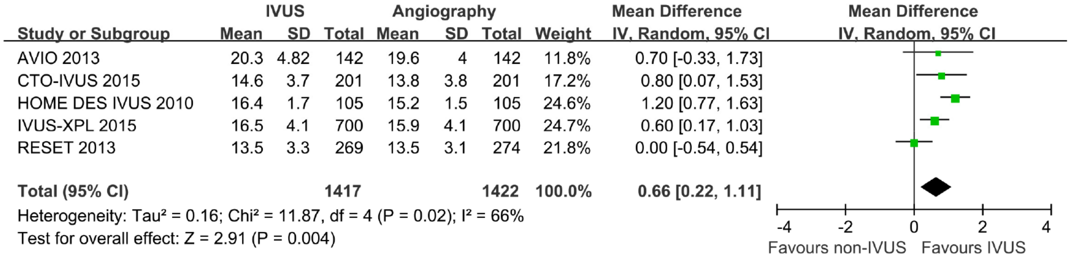

D. post-stent minimal lumen diameter

Study or Subgroup	IVUS Mean	IVUS SD	IVUS Total	Angiography Mean	Angiography SD	Angiography Total	Weight	Mean Difference IV, Random, 95% CI
AVIO 2013	2.7	0.46	142	2.51	0.46	142	10.1%	0.19 [0.08, 0.30]
CTO-IVUS 2015	2.64	0.35	201	2.56	0.41	201	14.0%	0.08 [0.01, 0.15]
HOME DES IVUS 2010	2.94	0.31	105	2.87	0.24	105	13.9%	0.07 [-0.00, 0.14]
IVUS-XPL 2015	2.64	0.42	700	2.56	0.39	700	18.5%	0.08 [0.04, 0.12]
RESET 2013	2.6	0.37	269	2.57	0.4	274	15.3%	0.03 [-0.03, 0.09]
Tan et al 2015	3.44	0.12	61	3.43	0.09	62	19.1%	0.01 [-0.03, 0.05]
Tian et al 2015	2.62	0.45	115	2.4	0.47	115	9.0%	0.22 [0.10, 0.34]
**Total (95% CI)**			**1593**			**1599**	**100.0%**	**0.08 [0.03, 0.13]**

Heterogeneity: Tau² = 0.00; Chi² = 21.44, df = 6 (P = 0.002); I² = 72%
Test for overall effect: Z = 3.42 (P = 0.0006)

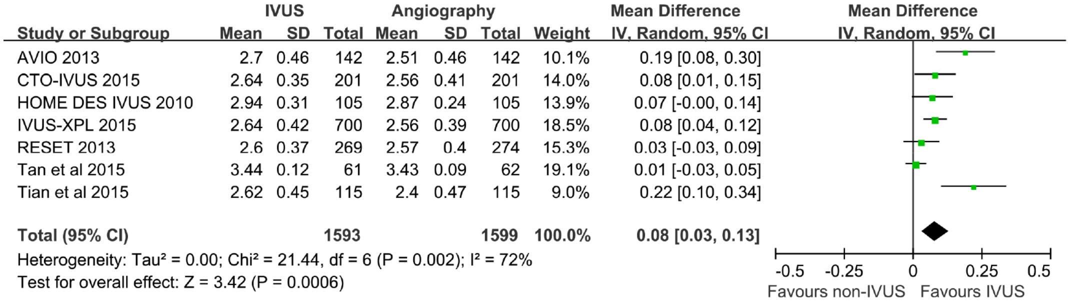

E. post-stent diameter stenosis

Study or Subgroup	IVUS Mean	IVUS SD	IVUS Total	Angiography Mean	Angiography SD	Angiography Total	Weight	Mean Difference IV, Random, 95% CI
AVIO 2013	8.4	7.9	142	10.5	9	142	11.0%	-2.10 [-4.07, -0.13]
CTO-IVUS 2015	9	9.8	201	10.2	10.9	201	10.4%	-1.20 [-3.23, 0.83]
HOME DES IVUS 2010	14.6	7.1	105	15.3	6.1	105	13.3%	-0.70 [-2.49, 1.09]
IVUS-XPL 2015	12.79	8.66	700	13.74	8.05	700	55.7%	-0.95 [-1.83, -0.07]
Tian et al 2015	10.96	5.66	115	13.76	10.12	115	9.5%	-2.80 [-4.92, -0.68]
**Total (95% CI)**			**1263**			**1263**	**100.0%**	**-1.25 [-1.90, -0.59]**

Heterogeneity: Tau² = 0.00; Chi² = 3.59, df = 4 (P = 0.46); I² = 0%
Test for overall effect: Z = 3.73 (P = 0.0002)

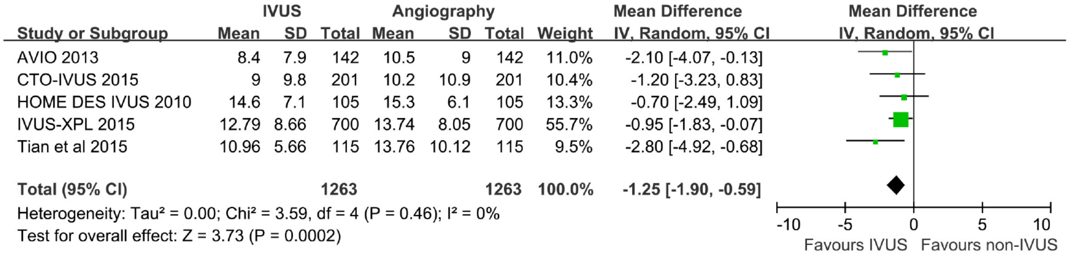

Supplementary Figure 2: Meta-analyses for main procedural parameters.

A. MACE

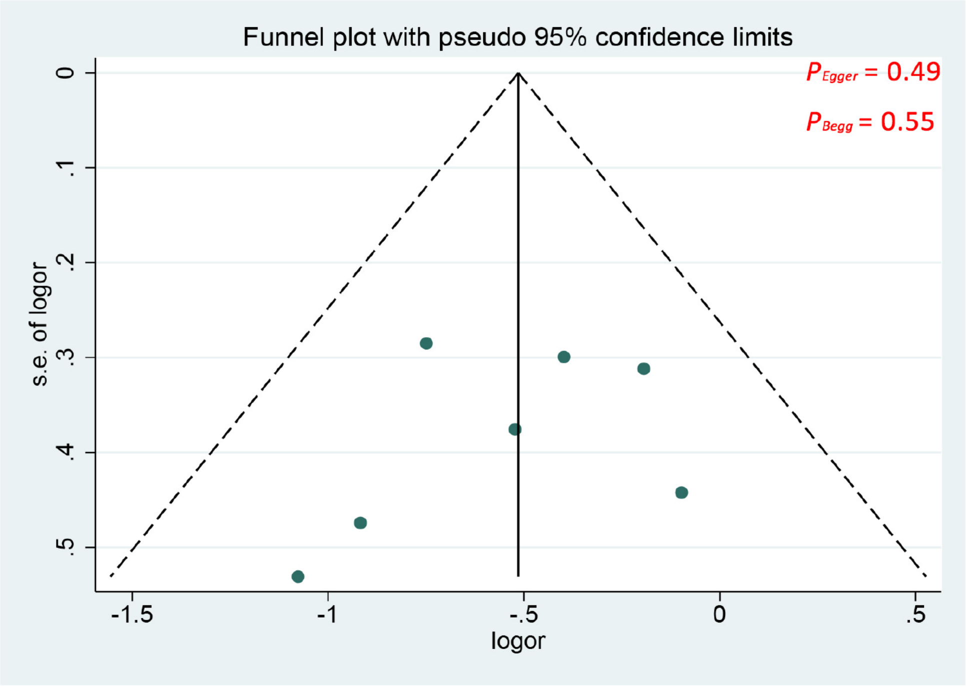

B. ST

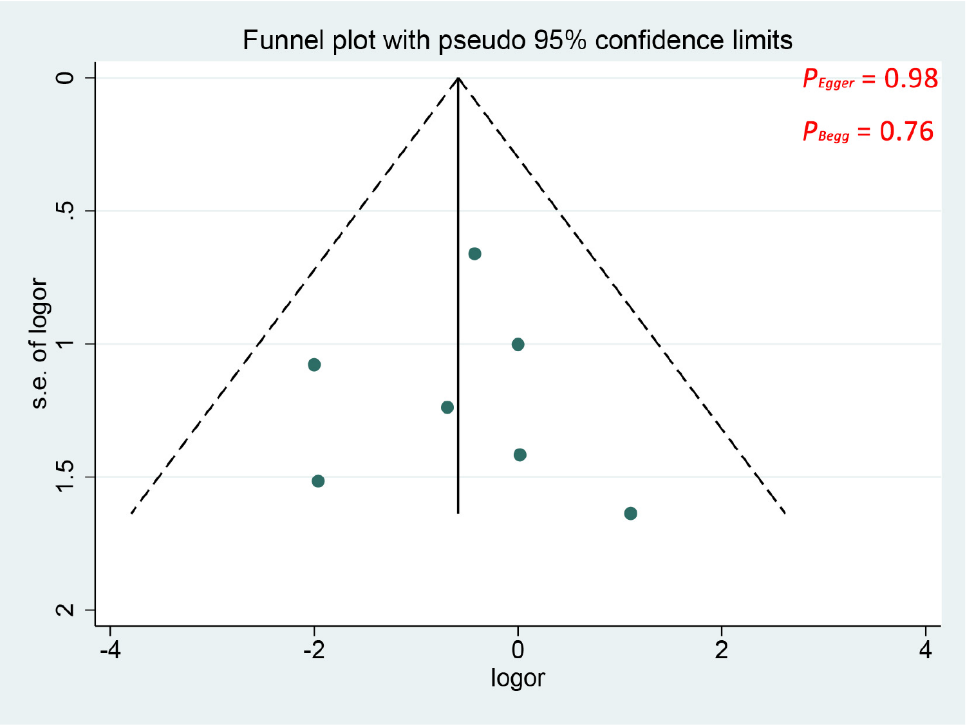

Supplementary Figure 3: Publication bias assessment for major adverse cardiac events **(A)** and stent thrombosis **(B)**.

Supplementary Table 1: Baseline angiographic and procedural characteristics

Tial	Target LAD (%)	Target LCX (%)	Target RCA (%)	Target LMCA (%)	RVD (mm)	MLD (mm)	DS (%)	lesion Length (mm)	Post-stent Dilation (%)	IVUS Criteria for Optimal DES Implantation	Post-stent DAPT
AVIO	53/49	NA	NA	NA	2.7/2.6	0.8/0.7	71.6/75.5	NA	88/66*	The AVIO criteria accoding to optimal balloon size	NA
CTO-IVUS	42/47	14/16	44/37	0/0	2.7/2.6	NA	NA	36.3/35.5	51/41	Minimal stent area ≥distal reference lumen area; stent area at CTO segment ≥5 mm^2 as far asvessel area permits; and complete stent apposition	Aspirin 100 mg/d and clopidogrel 75 mg/d ≥1 year
HOME DES IVUS	56/54	11/15	29/24	3/4	3.2/3.0	1.1/1	82.3/79.2	18.1/17.6	33/0	Good apposition; optimal stent expansion (with MSA of 5 mm^2) or CSA > 90% of distal reference lumen CSA for small vessel/ and no edge dissection (5-mm margins proximal and distal to the stent)	Aspirin ≥100 mg/d indefinitely and clopidogrel 75 mg/d for 6 months
IVUS-XPL	65/60	14/15	21/25	0/0	2.9/2.9	0.8/0.8	71.1/71.4	34.7/35.2	76/57	A minimal lumen CSA greater than the lumen cross-sectional area at the distal reference segments	Aspirin 100 mg/d and Clopidogrel 75 mg/d ≥6 months
RESET	62/68	15/13	23/20	0/0	2.8/2.8	1/0.9	NA	29.6/30.6	55/45	Adjunct high-pressure dilation was performed according to the discretion of operators based on the IVUS findings	Aspirin 100 mg/d indefinitely and clopidogrel 75 mg/d for 3 or 12 months
Tan et al	0/0	0/0	0/0	100/100	NA	1.9/1.9	NA	NA	38/15	Achieving successful expansion defined as lumen area 90% or greater of the average reference lumen area pre-intervention	Aspirin 300 mg/d for the first months and 100 mg/d afterward, and clopidogrel 75 mg/d ≥1 year
Tian et al	44/37	21/15	35/46	0/3	2.7/2.6	NA	NA	29/31	NA	Good apposition; MSA >80% of reference vessel area; symmetry index >70%; and no >Type B dissection	NA

AVIO, angiography versus IVUS optimization; CSA, corss sectional area; CTO, chronic total occlusion; DAPT, dual antiplatelet therapy; DES, drug-eluting stent; DS, diameter stenosis; IVUS, intravascular ultrasound; LAD, left anterior descending; LCX, left circumflex; LMCA, left main coronary artery; MLD, minimal lumen diameter; MPDP, maximal poststent dialation pressure; MSA, minimal stent area; NA, not applicable; RCA, right coronary arter; RVD, reference vessel diame *represents the percentage of lesions.

Supplementary Table 2: Detailed search strategy (PubMed database)

Search line	Search term	No. Hits
#1	intravascular ultrasound[MeSH Terms]	5333
#2	IVUS[Title/Abstract]	3423
#3	#1 OR #2	6431
#4	angiography[MeSH Terms]	208733
#5	drug-eluting stent*[MeSH Terms]	7023
#6	percutaneous coronary intervention[MeSH Terms]	39666
#7	#5 OR #6	42707
#8	(#3 AND #4) AND #7	766
#9	IVUS-guided [Title/Abstract]	221
#10	IVUS guidance[Title/Abstract]	162
#11	#9 OR #10	303
#12	drug-eluting stent*[Title/Abstract]	7760
#13	percutaneous coronary intervention[Title/Abstract]	19683
#14	#12 OR #13	25033
#15	#11 AND #14	128
#16	#8 OR #15	850
#17	#16 AND (“2001-01”[Date - Publication] : “2015-12”[Date - Publication])	732

Supplementary Table 3: Meta-regression analyses for MACE and ST

Covariable	MACE		ST	
	Coefficient	$P_{interaction}$	Coefficient	$P_{interaction}$
Acute coronary syndrome	-0.08	0.92	2.08	0.37
Diabetes	-0.79	0.77	2.12	0.75
Chronic total occlusion	0.19	0.58	-1.97	0.14
New-generation DES	-0.40	0.31	0.20	0.84
Mean baseline age	-0.02	0.57	-0.03	0.75
Stent length	0.003	0.24	-0.05	0.26

DES, drug-eluting stents; MACE, major adverse cardiac events; ST, stent thrombosis.
